# Online Comprehensive Care Therapy Complementary Program Improves Quality of Life in People with Parkinson’s Disease

**DOI:** 10.3390/brainsci16090906

**Published:** 2026-08-25

**Authors:** Diego Santos García, Pablo Campo Prieto, Carmen M. Breijo García, Lucía Dafonte Gil, Juan Pereiro Nogueira, Jessica Blanco López, Francisco Doblas

**Affiliations:** 1 Department of Neurology, Hospital Universitario de A Coruña (HUAC), Complejo Hospitalario Universitario de A Coruña (CHUAC), 15006 A Coruña, Spain; 2 Grupo de Investigación en Enfermedad de Parkinson y otros Trastornos del Movimiento, Instituto de Investigación Biomédica de A Coruña (INIBIC), 15006 A Coruña, Spain; 3 Department of Neurology, Hospital San Rafael, 15006 A Coruña, Spain; 4 Fundación Degen, 15006 A Coruña, Spain; pcampo@uvigo.gal (P.C.P.); carmenbreijo@gmail.com (C.M.B.G.); mar659_@hotmail.com (J.B.L.);; 5 Networking Research Center on Neurodegenerative Diseases (CIBERNED), Instituto de Salud Carlos III, 28029 Madrid, Spain; 6 Departamento de Bioloxía Funcional e Ciencias da Saúde, Facultade de Fisioterapia, Universidade de Vigo, 36310 Pontevedra, Spain; 7 Grupo de Investigación HealthyFit, Instituto de Investigación Sanitaria Galicia Sur (IISGS), SERGAS-UVIGO, 36312 Vigo, Spain; 8 Centro de Psicología Carmen Breijo, 15160 Sada, Spain

**Keywords:** mood, online, physiotherapy, Parkinson’s disease, quality of life, speech therapy

## Abstract

**Highlights:**

**What are the main findings?**

- A novel multidisciplinary online program simultaneously integrated five non-pharmacological therapeutic domains for people with Parkinson’s disease.
- After 6 months, the intervention significantly improved health-related quality of life, with a 12.9% reduction in the PDQ-39 total score, compared with 1.5% in controls.
- The program also significantly improved mood and reduced the non-motor symptom burden in treated participants.

**What are the implications of the main findings?**

- High adherence and the absence of reported adverse events support the feasibility and safety of comprehensive online care in Parkinson’s disease.

**Abstract:**

**Objectives:** Non-pharmacological therapies are a cornerstone of Parkinson’s disease (PD) treatment, but there is not enough evidence of their benefit when delivered altogether online. Our objective was to analyze the efficacy and safety of an online comprehensive care therapy program (OL-CCTP) in people with PD (PwP). **Material and methods:** This was a proof-of-concept experimental, prospective, 6-month interventional study comparing a PD treatment group (PwP-T) with a control group (PwP-C). The OL-CCTP included group sessions of physical therapy/therapeutic exercise, nutrition, speech therapy, cognitive stimulation, and psychological support (126 sessions over 6 months). The primary outcome was the change in quality of life at 6 months, as measured by the PDQ-39 questionnaire. Mood (Beck Depression Inventory-II [BDI-II]), the non-motor symptom burden (Non-Motor Symptoms Scale [NMSS]), and disability (Schwab and England Activities of Daily Living Scale [ADLS]) were also assessed. **Results:** Sixty patients (36 PwP-T and 24 PwP-C) completed the assessments. A reduction of 12.9% (from 78.4 ± 17.9 to 68.3 ± 18.7; *p* < 0.0001) was observed in the PDQ-39 total score in the PwP-T group (*p* < 0.0001), compared to 1.5% (from 77.8 ± 22.2 to 76.6 ± 27.9; *p* = 0.438) in the PwP-C group (*p* = 0.009). The BDI-II and NMSS scores decreased significantly by 13.1% (*p* = 0.001) and 20.3% (*p* = 0.046), respectively, in the PwP-T group but not in the PwP-C group. There were no significant changes in ADLS in either group. No adverse events were reported. **Conclusions:** After 6 months of OL-CCTP, an improvement of 12.9% was observed in the quality of life of patients with PD.

## 1. Introduction

Parkinson’s disease (PD) is a complex disorder in which there is not only a dopamine deficiency that causes motor symptoms but also deficiencies in other neurotransmitters, resulting in the development of non-motor symptoms (NMS) such as depression, anxiety, apathy, pain, fatigue, or cognitive impairment [1]. Pharmacological treatment for PD is symptomatic, with levodopa and other dopaminergic therapies being shown to improve motor symptoms, NMS, and quality of life (QoL) [2]. However, in the management of people with PD (PwP), other complementary therapies that have shown a benefit, such as physical exercise, physiotherapy, or speech therapy, are also important. Exercise is now an integral part of PD treatment, with evidence supporting aerobic, resistance, and balance training as effective physical therapy [3]. Physical therapy reduces disability and improves QoL, with early referral recommended to establish an evidence-based program [4]. Speech therapy (including the LSVT LOUD program) improves voice, speech intelligibility, and swallowing, with benefits maintained for up to 2 years post-treatment [5]. In addition to the above, it is important to highlight the importance of adequate emotional support and proper nutrition [6]. In this context, a multidisciplinary approach integrating occupational therapy, physical therapy, speech language pathology/therapy, and psychology/neuropsychology is recommended across all stages of the disease [7]. However, a problem for many patients is access to these therapies. They are often not covered by healthcare systems and are administered by experts from PD patient associations. Given that many patients live far from an association where they can receive these therapies, an alternative would be to conduct them online.

A recent systematic review confirmed that telerehabilitation physiotherapy interventions were at least as effective as traditional rehabilitation, with high patient satisfaction and adherence equivalent to in-person care [8]. e-Exercise is optimal for motor function and physical performance, while e-cognitive care is most effective for psychological and cognitive challenges [9]. Multiple randomized clinical trials demonstrate the non-inferiority of speech therapy/LSVT LOUD online compared to face-to-face therapy for the treatment of hypokinetic dysarthria [10]. Moreover, telerehabilitation is considered safe, with most reported adverse events being rare, non-serious or mild, and unrelated to telerehabilitation protocols [11]. The main advantages of telerehabilitation are access for rural patients, reduced travel costs, and improved adherence. On the other hand, the main current limitations are the scarcity of studies with large samples, the lack of standardized protocols, and the need for more multidisciplinary studies [12]. In summary, these findings highlight the importance of tailoring telemedicine programs with a multidisciplinary approach to address specific therapeutic needs in PD management.

A comprehensive care system for PwP, based on specialized care (psychology, physiotherapy, nutrition, and speech therapy), can be organized into a single program that centralizes and coordinates the different therapeutic actions and delivers them through virtual group sessions. This would reduce the management complexity and costs for patients. We hypothesize that receiving comprehensive online therapy, compared to not doing so, will have a positive impact on the QoL of patients with PwP. In this context, the aim of this study was to evaluate the effects on the QoL of PwP of an online comprehensive care therapy program (OL-CCTP) based on five axes that included physical therapy/therapeutic exercise, nutrition, speech therapy, cognitive stimulation, and psychological support applied online. Specifically, a group of PwP treated (PwP-T) with this program was compared in terms of efficacy with a group of PwP who did not receive the treatment (control group; PwP-C).

## 2. Material and Methods

This was a prospective proof-of-concept experimental study with 2 randomized arms (2:1) consisting of intervention (PwP-T) versus no intervention (PwP-C), with prospective follow-up for 6 months, conducted in PwP. The inclusion criteria were a diagnosis of PD according to the 2015 MDS criteria [13], based on a medical report issued by the neurologist who was monitoring the patient; the capacity to freely decide to participate; Hoehn and Yahr stage (H&Y) 1 to 3; a stable dose of PD medication and no anticipated changes in it from baseline throughout the study duration based on the clinical status during screening and prior to randomization; availability for follow-up during the study period (6 months); and signing of an informed consent form. PwP with the following criteria were excluded: another cause of parkinsonism other than PD; cognitive impairment and/or dementia affecting comprehension and appropriate decision-making; active psychosis with hallucinations and/or delusions; severe major depression refractory to medication; H&Y 4 or 5; total dependence for basic activities of daily living; inability to participate in follow-up; comorbidities that limited the possibility of carrying out the intervention; already actively receiving comprehensive therapy in person at a patient association or other setting; another justified reason, at the discretion of the coordinating investigator (DSG), that precluded participation in the therapy.

Patient enrollment was conducted through the Degen Community website. This website is an information channel offering various services for PwP and has been operational since 28 February 2025 (https://www.fundaciondegen.org/comunidad/, accessed on 24 August 2026). The Degen Foundation is the entity responsible for this domain. If a patient wished to participate, he/she had to complete an initial form with data related to sociodemographic aspects and factors related to PD. Subsequently, the information was reviewed by D.S.G., as a neurologist and expert on PD, and the patient’s eligibility was confirmed. Participants were not specifically assessed by D.S.G. face-to-face. Participants were randomized to the intervention (PwP-T) or control (PwP-C) group at a 2:1 allocation ratio using a permuted block randomization procedure with a fixed block size of three participants. Within each block, two participants were allocated to the intervention group and one to the control group, with the order of assignments randomly permuted. The allocation sequence was generated and retained exclusively by an external member of the project team (F.D.), who was not involved in participant recruitment, eligibility assessment, clinical evaluations, or the delivery of the intervention. The investigators responsible for recruitment and eligibility assessment had no access to the allocation sequence, and the corresponding assignment was communicated only after eligibility had been confirmed and the participant had been formally enrolled. No computerized central randomization system or sequentially numbered opaque sealed envelopes were used. The participant recruitment period lasted 3 months (from April to June 2025).

**Figure 1:**
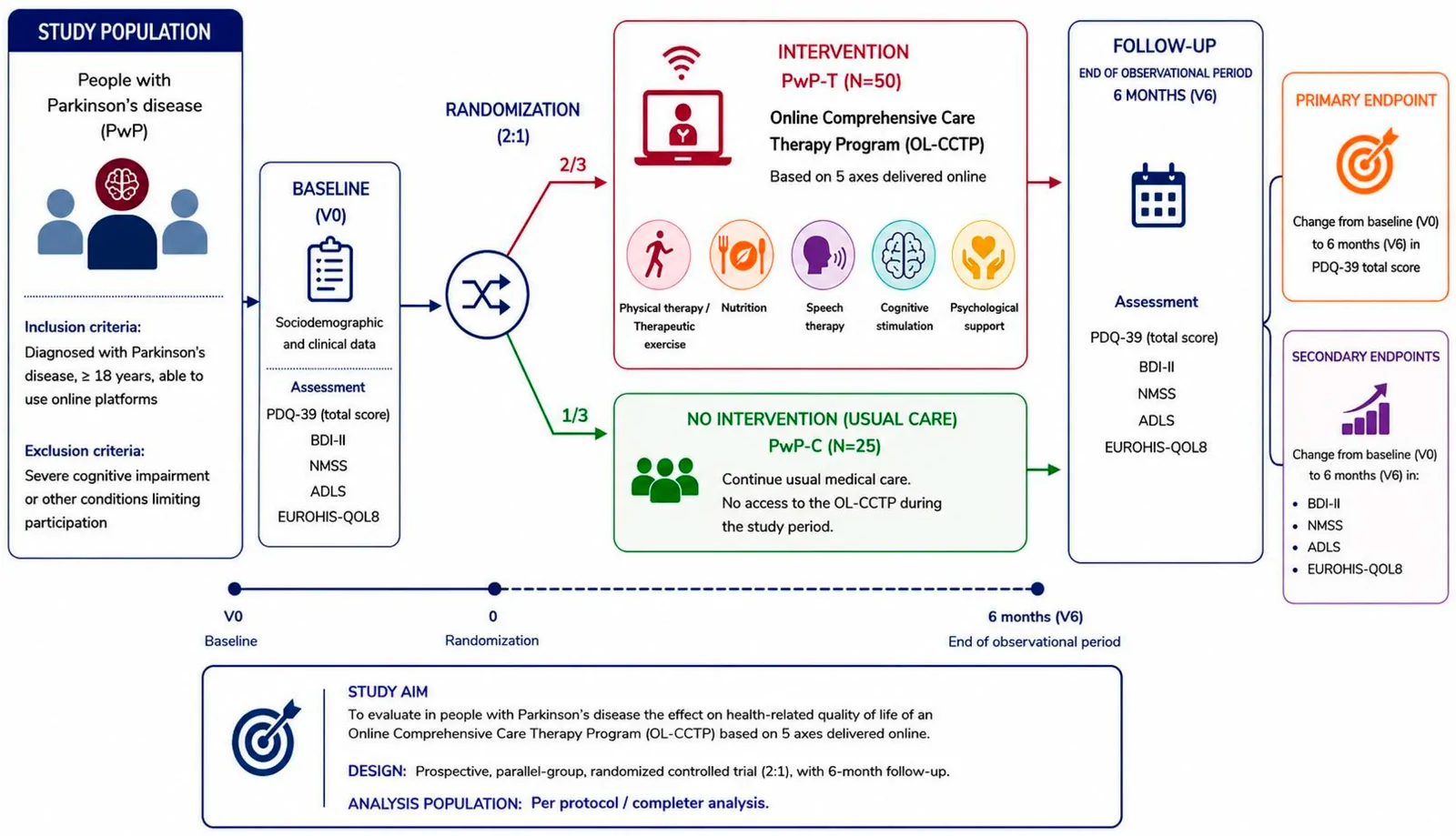
Study protocol with description of the two arms (intervention vs. control) and therapies performed during 6-month follow-up.

PwP who received the OL-CCTP (PwP-T) were divided into groups of 9–12 participants according to their characteristics and clinical status. The OL-CCTP included online group sessions for psychological support and cognitive stimulation with one session per week, with a total of 24 sessions over 6 months; online group exercise sessions (multicomponent exercise program with aerobic exercise, strength–power, balance, coordination, and flexibility) with two sessions per week, with a total of 48 sessions over 6 months; online group nutrition sessions with one session per month, with a total of 6 sessions over 6 months; and online group speech therapy sessions with a maximum of two sessions per week, depending on the specific needs of each patient, with a maximum of 48 sessions over 6 months. Overall, the total number of therapies received across 6 months per patient reached a maximum of 126 (24 + 48 + 6 + 48), with each one lasting about 1 h (Figure 1). All therapies were implemented by professionals who were experts in each of the interventions: C.M.B.G., a psychologist; L.D.G., a physiotherapist and rehabilitation specialist in therapeutic exercise; J.P.N., a nutrition expert; and J.B.L., a speech therapist.

Participants completed a series of validated scales at baseline before randomization (V0) and subsequently every month up to 6 months (±15 days) of follow-up (V6): the Beck Depression Inventory-II (BDI-II) [14] to assess depressive symptoms; the Non-Motor Symptoms Scale (NMSS) [15] to assess NMS; the 39-item Parkinson’s Disease Questionnaire (PDQ-39) [16] to assess health-related QoL; the EUROHIS 8-item questionnaire (EUROHIS-QOL8) [17] to assess global QoL; and the Schwab and England Activities of Daily Living Scale (ADLS) [18] to assess disability.

### 2.1. Statistical Analysis

Data were processed using SPSS 20.0 (IBM, Chicago, IL, USA) for Windows. Different variables were expressed as quantitative and/or qualitative variables. The distributions of variables were verified by the one-sample Kolmogorov–Smirnov test. The primary efficacy outcome was the change from baseline (V0) to the end of the observational period (V6) in the PDQ-39 total score. There were 39 items grouped into 8 domains: (1) “mobility” (items 1 to 10); (2) “activities of daily living” (items 11 to 16); (3) “emotional well-being” (items 17 to 22); (4) “stigmatization” (items 23 to 26); (5) “social support” (items 27 to 29); (6) “cognition” (items 30 to 33); (7) “communication” (items 34 to 36); and (8) “bodily discomfort” (items 37 to 39). For each item, the score may range from 0 (never) to 4 (always). The symptoms referred to the 4 weeks prior to assessment. The total score ranged from 0 to 156 (maximum, 39 × 4). Every domain was expressed as a percentage to be able to perform comparisons. Secondary efficacy measures were the change from V0 to V6 in the total score on the BDI-II, NMSS, EUROHIS-QOL8, and ADLS.

Given the proof-of-concept nature of the study, no formal hypothesis-driven sample size calculation was performed. A target sample of 75 participants was considered feasible and adequate to provide preliminary estimates of the efficacy and safety of the intervention. Participants were planned to be randomized at a 2:1 ratio, corresponding to 50 participants in the intervention group (PwP-T) and 25 in the control group (PwP-C). This sample size also took into account an anticipated rate of approximately 20–30% of participants not being evaluable for the primary efficacy analysis because of insufficient adherence, incomplete assessments, or other eligibility or follow-up issues.

The efficacy analysis was performed on those participants who had adherence ≥80% in the OL-CCTP (PwP-T) and ≥80% [19] in the completion of the questionnaires (PwP-T and PwP-C), with all questionnaires being completed at V0 and V6 also mandatory. Firstly, a Wilcoxon’s rank sum test was performed to test the change from V0 to V6 in each group, PwP-T and PwP-C. To evaluate the magnitude of the change, in addition to the difference between V0 and V6, the relative change [RC = (mean Test_V6_ − mean Test_V0_) × 100/mean Test_V0_] [20] and Cohen’s d_av_ effect size [ES = (mean Test_V6_ − mean Test_V0_)/SD_pooled_)] [21] were calculated. The values were considered as follows: negligible, <0.2; small, 0.2–<0.5; moderate, 0.5–<0.8; large, 0.8–1.3; very large, ≥1.3. Secondly, a repeated-measures ANOVA was performed to test the “group × time” interaction between the group that received the intervention (PwP-T) and the control group (PwP-C) at post-treatment compared to pre-treatment, unadjusted and adjusted for covariates (age, sex, and disease duration). The change in a variable was considered to show a significant difference when the *p*-value was <0.05. The study was considered to have achieved the main objective when the change in PDQ-39 from V0 to V6 (primary endpoint) in the PwP-T group showed a significant reduction with *p* < 0.05 for the three proposed analyses (Wilcoxon’s rank sum and repeated-measures ANOVA). Correlations between the change in health-related QoL (PDQ-39) from V0 to V6 and the changes in other continuous variables from V0 to V6 (BDI-II; NMSS; ADLS; EUROHIS-QOL8) in both groups were analyzed using Pearson or Spearman correlation coefficients, depending on the data distribution. Correlations were interpreted as weak (≤0.29), moderate (0.30–0.59), or strong (≥0.60). The estimated sample size was 75 participants (50 in the PwP-T group and 25 in the PwP-C group) with an estimated 20 to 30% of failure or selection problems and/or inadequate completion of the study.

Regarding the safety analysis, and considering the type of intervention, only potential adverse events directly or indirectly related to the therapy were recorded at the discretion of the therapy team. These could have been physical, emotional, or of a different nature. The safety dataset consisted of all subjects for whom the study was initiated.

### 2.2. Standard Protocol Approvals, Registrations, and Patient Consent

For this study, we received approval from the Comité de Ética de Investigación de Medicamentos de Galicia (CEImG) in Spain (2025/131; 24 April 2025). Written informed consent was obtained from all participants in this study.

### 2.3. Data Availability

The protocol and the statistical analysis plan are available on request. Deidentified participant data are not available for legal and ethical reasons.

## 3. Results

A total of 60 out of 75 PD patients were included and valid for the analysis (80% of the proposed sample). Of these, 36 PD patients received the intervention (PwP-T) (72% of the proposed sample), whereas 24 were in the control group (PwP-C) (96% of the proposed sample). The reason for excluding 14 patients from the PwP-T was insufficient adherence to the questionnaires. All patients who received the intervention and were finally included in the analysis had adherence to the online sessions of greater than 90%. One control subject was also excluded for not completing the questionnaires properly. The mean age was 58.3 ± 8.7 years old, and 68.3% were women. About three out of four patients were from Spain, with the rest from other countries (Argentina, Ecuador, Mexico, Colombia, Peru, Nicaragua, and the United Kingdom). Only 11.7% of patients had a principal caregiver (Table 1). The three most frequent disabling symptoms reported by the patients were bradykinesia (38.3%), rigidity (38.3%), and tremor (28.3%). Regarding baseline sociodemographic and PD-related characteristics, no differences were detected between the two groups (Table 1).

**Figure 2:**
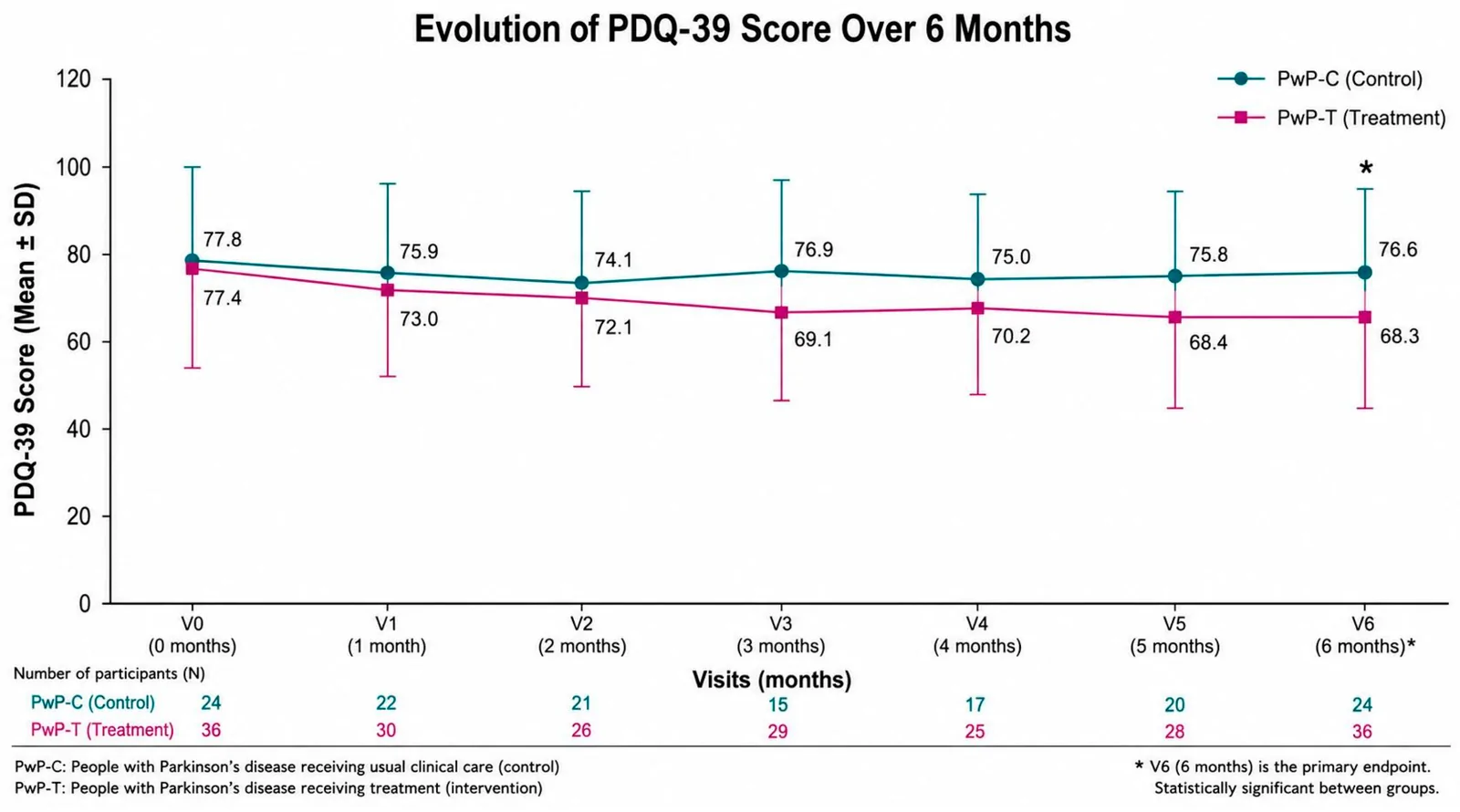
Change in the PDQ-39 total score from baseline visit (V0) to the end of the intervention period (V6) over 6 months of follow-up in both groups of PwP, i.e., treated (PwP-T) and controls (PwP-C). * Significance (*p* < 0.05) at V6 (primary endpoint).

From baseline (V0) to the final observational period (V6), the PDQ-39 total score decreased significantly from 78.4 ± 17.9 to 68.3 ± 18.7 (RC = −12.9%; *p* < 0.0001) in the PwP-T group but not in the PwP-C group (from 77.8 ± 22.2 to 76.6 ± 27.9; RC = −1.5%; *p* = 0.438) (Table 2 and Figure 2). The difference in the change from V0 to V6 in the PDQ-39 between groups (PwP-T vs. PwP-C) was significant without (*p* = 0.011) and after adjusting for covariates (*p* = 0.009). By domain, a significant improvement was observed in the PwP-T group in mobility (RC = −9.1%; *p* = 0.038), emotional well-being (RC = −20.6%; *p* < 0.0001), stigma (RC = −20.6%; *p* = 0.001), cognition (RC = −9.6%; *p* = 0.026), communication (RC = −17.8%; *p* = 0.002), and bodily discomfort (RC = −12.9%; *p* = 0.029) (Table 2). The largest effect, according to the ES, was detected for communication (ES = 2.17), followed by emotional well-being (ES = 2.15). After adjustment for multiple comparisons using the Bonferroni correction (adjusted significance threshold, *p* < 0.00625), only the improvements in emotional well-being, stigma, and communication remained statistically significant. Other domain-level findings with nominal *p* values <0.05 should therefore be considered exploratory. No improvement was detected for any domain of the PDQ-39 in the PwP-C group. In the repeated-measures ANOVA, a significant difference indicating an improvement in activities of daily living, emotional well-being, communication, and bodily discomfort was detected in favor of the PwP-T group compared to the PwP-C group (Table 2). At the end of follow-up (V6), 13.9% (5/36) of PwP who received the intervention (PwP-T) had a higher score on the PDQ-39 questionnaire, compared to 33.3% (8/24) in the control group (PwP-C).

Regarding the secondary variables, an improvement was observed in the Pw-P-T group in mood and NMSS burden, with a decrease from V0 to V6 in the BDI-II and NMSS total scores from 13.7 ± 7.3 to 11.9 ± 16.1 (RC = −13.1%; *p* = 0.001) and from 47.2 ± 31.4 to 37.6 ± 28.0 (RC = −20.3%; *p* = 0.046), respectively. No significant change was detected in the treated group in either autonomy for activities of daily living (ADLS) or global QoL (EUROHIS-QOL8) (Table 2). In the PwP-C, no significant changes were observed from V0 to V6 in any of the secondary variables (Table 2). A strong correlation between the improvement detected in PwP-T from V0 to V6 in health-related QoL (PDQ-39) and mood (BDI-II) was found (r = 0.609; *p* < 0.0001), whereas moderate correlations were found between the change from V0 to V6 in the PDQ-39 total score and the scores on the NMSS, ADLS, and EUROHIS-QOL8 (Table 3). In the repeated-measures ANOVA, no differences in the change from V0 to V6 in any secondary variable between the two groups, PwP-T vs. PwP-C, were detected (Table 2).

Regarding side effects, no adverse events (e.g., falls, fainting, injuries, etc.) were observed by therapists and/or reported by patients during the intervention procedure (at the time). Moreover, no patient reported any adverse events afterward that they believed were due to the intervention (e.g., pain resulting from excessive muscle activity, dizziness, confusion, etc.).

## 4. Discussion

This study observed that a multidisciplinary, non-pharmacological intervention (physical therapy/therapeutic exercise, nutrition, speech therapy, cognitive stimulation, and psychological support), completed online by PwP, contributed to an improvement in their perceived QoL. In particular, the primary endpoint of the study was met despite the fact that it was an experimental study with a small sample size. Although a large, comprehensive randomized controlled trial would be necessary, to our knowledge, these findings are novel, since this is the first study to apply an online therapy based on five axes simultaneously (physical therapy/therapeutic exercise + nutrition + speech therapy + cognitive stimulation + psychological support). Specifically, there is a substantial and growing body of evidence for online/telerehabilitation in PD, with studies covering these domains either individually or in various combinations, but a trial with all aspects at the same time has not yet been published [22]. This is particularly relevant given the high percentage of patients with limited access to these complementary therapies.

Other complementary therapies to pharmacological treatment have been shown to improve the clinical status and QoL of PwP [4,5,6,7,8,9,10]. In fact, some non-pharmacological interventions are now beginning to be included in therapy guidelines [23]. However, one of the problems is access to these treatments. In Spain, they are often provided through patient associations, but only an estimated 6% to 10% of PwP in Spain actively access or belong to formal patient associations. The online use of these complementary therapies eliminates the barrier regarding the time needed to travel to distant locations, and, importantly, they are applied by qualified professionals and in an appropriate manner. In addition, it is of interest that, in our study, women represented nearly 70% of the sample. While PD affects roughly 1.5 times more men than women, extensive health informatics and behavioral research demonstrate that women are more proactive as online health information seekers. For women living with PD, this digital resource-seeking behavior is driven by specific clinical, social, and caregiving disparities [24,25]. Another important point is age, with the participants in our study being younger, with an average age of just under 60 years. PwP under 65 are more likely to use internet sources to acquire disease-specific knowledge and connect with digital health communities, whereas older PwP (>65 years old) generally use digital tools less frequently, instead prioritizing direct communication with neurologists or healthcare professionals for health guidance [26]. Obviously, this poses a potential limitation when considering online treatment for older people, and studies specifically conducted on elderly individuals are needed. Also noteworthy is the high PDQ-39 total score found at baseline, with an average of 78 points, equivalent to 50 on the PDQ-39SI (Summary Index). The higher participation of women, who are known to tend to score higher [27], may have contributed to this, along with a possible bias in the participation of patients who subjectively felt worse and required complementary therapies despite being young and having a relatively short disease duration. Furthermore, the questionnaires were self-administered.

In our study, we observed that PwP who received, over 6 months, an OL-CCTP that included physical therapy/therapeutic exercise, nutrition, speech therapy, cognitive stimulation, and psychological support achieved improvements in health-related QoL, mood, and NMS burden, which was not observed in PwP who did not receive the intervention. In particular, activities of daily living, emotional well-being, communication, and bodily discomfort related to QoL perception improved in the treated PD patients as compared to those without the intervention. Multidisciplinary rehabilitation (physical therapy/therapeutic exercise speech therapy, occupational therapy, psychology/neuropsychology) is strongly endorsed by guidelines as an essential, non-pharmacological pillar of PD management across all disease stages [4] and can improve QoL for individuals with PD [28]. A 2023 Cochrane systematic review and network meta-analysis (156 RCTs) confirmed the beneficial effects of most physical exercise types on both motor severity and health-related QoL [29]. Telerehabilitation (online delivery) has been shown to produce outcomes comparable to those of in-person therapy for PD patients, with benefits in motor function, QoL, mood, activities of daily living, and cognition [22]. A 2025 systematic review and network meta-analysis (23 RCTs, n = 1330) found that e-exercise produced the greatest motor improvements and e-cognitive interventions yielded the greatest cognitive and psychological gains [9]. The optimal telerehabilitation dosing is approximately 1 h/day, 3–4 days/week, for 4–12 weeks, being particularly beneficial in early-stage PD with preserved cognition [30], in line with the intervention carried out in our study if we take into account the other activities complementary to physical therapy. In comparison with other previous studies, and as a novelty of the present approach, in our study, the online intervention was carried out at multiple levels, not only working on the physical state but also the cognitive, emotional, and communication levels. The vCare trial [31] is the closest existing study to a multi-intervention, with an online multidomain virtual training platform (ICT-based) for PD addressing motor function, cognition (MoCA), QoL, and autonomy for activities of daily living. The PD group who received the intervention exhibited significantly improved QoL, cognition, motor symptoms, and daily life activities compared to the control group, but only 10 patients were included in each group. Online/telehealth speech therapy (notably Lee Silverman Voice Treatment delivered via videoconferencing) has RCT evidence of non-inferiority to face-to-face delivery in PD [32]. Online cognitive telerehabilitation in PD has RCT evidence for improvements in cognitive performance [33]. Online psychological interventions (CBT by telephone) have been tested in an RCT format in PD, showing improvements in depression and anxiety [28]. Nutrition delivered online as part of a multimodal PD program is the least-studied component and is the most notable gap in the online multimodal literature [34]. In this context, the Movement Disorder Society (MDS) Telemedicine Study Group has an active subgroup specifically developing recommendations for online non-pharmacological multimodal rehabilitation in PD, and many other diverse expert proposals are underway [35], whereas the Spanish Society of Neurology (SEN) 2024 Parkinson Guideline addresses telemedicine in PD management, noting high patient satisfaction with remote consultations, alongside some concerns [36]. The fact that aspects of QoL such as the perception of emotional well-being, activities of daily living, and communication improved in our study as compared to not receiving the intervention suggests the importance of a multi-intervention approach to different symptoms of the disease. Although no differences were observed between groups in the change in mood stage (BDI-II), it did improve in the treated group, and its improvement was correlated with the improvement in QoL, which is known and reported in PD [37]. The correlation of changes in health-related QoL with the other aspects of the disease (mood, NMS burden, autonomy, and overall QoL) gives consistency to the findings and highlights the possibility of a bidirectional relationship with the influence of mood—that is, an improvement in symptoms positively impacts the perception of QoL and mood, and a better mood leads to better perceived QoL and a more optimistic attitude when answering the questionnaires. Furthermore, telemedicine can not only be effective in improving certain symptoms in PwP but also cost-efficient [38].

The present study has some limitations. First, the sample size of the groups was small and differed between treated patients (N = 36) and controls (N = 24). Furthermore, a proportion of the participants allocated to the intervention group were not included in the final efficacy analysis due to insufficient adherence to questionnaire completion, which may have introduced selection bias. However, they were comparable in that no differences were detected in sociodemographic and PD-related aspects (PDQ-39, BDI-II, NMSS, ADLS, and EUROHIS-QOL8) at baseline. Second, the intervention time with subsequent evaluation was limited to 6 months, but the delayed effect of the therapy after stopping the intervention was not assessed. Third, this was not a blind study with respect to the intervention, which is common in this type of study, so there could have been an added placebo effect. Furthermore, although the allocation sequence during randomization was retained by an external team member and was not disclosed until participant enrollment, the use of a fixed block size of three without a formal centralized allocation concealment system may have introduced some allocation predictability and selection bias. Fourth, the different items of the NMSS and BDI-II scales were not analyzed, focusing only their total scores. In addition, given the multiple comparisons performed across the PDQ-39 domains, some nominally significant findings did not remain significant after Bonferroni correction and should therefore be interpreted with caution. Fifth, some assessments, such as a cognitive test, weight, or other biological markers, were not evaluated. Sixth, although PD patients at baseline had to be on a stable regimen of PD medication without anticipated changes in it from baseline throughout the study, the dopaminergic medication type, doses, and changes were not collected. Seventh, since all outcomes were self-reported and the study was not blinded, it cannot be ruled out that part of the observed improvement (particularly in the PDQ-39 emotional well-being domain and the BDI-II) reflects non-specific effects related to social support or attention, rather than the specific therapeutic components of the intervention. Finally, some minor side effects of the intervention (fatigue, muscle soreness, etc.) not reported by patients cannot be ruled out.

## 5. Conclusions

In conclusion, this randomized study, comparing a simultaneous, multifaceted online intervention based on five axes versus no intervention, observed an improvement in QoL after 6 months. This study highlights the importance of a multidisciplinary approach to PD with non-pharmacological therapies and the potential of telerehabilitation to facilitate its use in clinical practice. Further studies along these lines with a larger number of patients are needed.

## Figures and Tables

**Table 1:** Sociodemographic and PD-related aspects of patients who finally participated in the study and completed the visits adequately (N = 60).

	PwP-C (N = 24)	PwP-T (N = 36)	Entire Cohort (N = 60)	*p*
Age	58.8 ± 7.9	58.0 ± 9.4	58.3 ± 8.7	0.732
Gender (women) (%)	62.5	72.2	68.3	0.304
Disease duration (years)	5.5 ± 5.0	5.2 ± 4.0	5.7 ± 5.7	0.704
Country (%)				
- Spain	79.2	75	76.7	0.312
- Another country	20.8	25	23.3	
Employment situation (%) ^1^				0.588
- Currently employed	45.8	32.3	38.2	
- Retired	29.2	35.5	32.7	
- Emerging but not working	25	32.3	29.1	
Principal caregiver (%)	4.2	16.7	11.7	0.117
Most relevant PD symptoms (%) ^2^				
- Tremor	33.3	25	28.3	0.339
- Rigidity	29.2	44.4	38.3	0.179
- Bradykinesia	37.5	38.9	38.3	0.566
- Axial symptoms	20.8	30.6	26.7	0.299
- Speech problems	8.3	13.9	11.7	0.412
- Fluctuations/off episodes	12.5	0	5	0.059
- Dyskinesia	12.5	5.6	8.3	0.311
- Neuropsychiatric symptoms	4.2	13.9	10	0.220
- Sleep/fatigue	29.2	22.2	25	0.377
- Dysautonomia symptoms	29.2	11.1	18.3	0.077
- Pain and sensorial symptoms	16.7	13.9	15	0.522
- Cognitive problems	8.3	2.8	5	0.350
PDQ-39	77.8 ± 22.2	78.4 ± 17.9	78.2 ± 19.6	0.920
BDI-II	12.7 ± 7.9	13.7 ± 7.3	13.3 ± 7.4	0.978
NMSS	56.0 ± 41.9	47.2 ± 31.4	50.8 ± 35.9	0.213
ADLS	77.5 ± 20.7	80.0 ± 17.1	79.0 ± 18.5	0.541
EUROHIS-QOL8	3.7 ± 0.7	3.7 ± 0.8	3.7 ± 0.8	0.991

The results represent % or mean ± SD (range) or median [p25, p75]. ^1^ N = 56 (for the rest of the variables, N = 60). ^2^ Symptoms reported as the most disabling for the patient in an open-ended questionnaire; chi-squared and Mann–Whitney–Wilcoxon tests were used. ADLS, Schwab and England Activities of Daily Living; BDI-II, Beck Depression Inventory II; EUROHIS-QOL8, EUROHIS 8-item questionnaire; NMSS, Non-Motor Symptoms Scale; PD, Parkinson’s disease; PDQ-39, 39-item Parkinson’s Disease Quality of Life Questionnaire. PwP, people with Parkinson’s disease; PwP-C, PwP control group; PwP-T, PwP treatment group.

**Table 2:** Change from baseline (pre-therapy; V0) to the final observational period (V6) in the primary endpoint (PDQ-39 total score) and its domains, as well as other secondary variables, in the PwP-C group (N = 24) compared to the PwP-T group (N = 36).

	PwP-C V0	PwP-C V6	PwP-C RC (%)	PwP-C ES	P^1^	PwP-T V0	PwP-T V6	PwP-T RC (%)	PwP-T ES	P^2^	P^3^	P^4^
**Primary endpoint**												
PDQ-39	77.8 ± 22.2	76.6 ± 27.9	−1.5	0.03	0.438	78.4 ± 17.9	68.3 ± 18.7	−12.9	0.37	**<0.0001**	0.011	**0.009**
- Mobility	54.1 ± 24.2	52.6 ± 22.7	−2.8	0.11	0.621	48.3 ± 17.4	43.9 ± 18.4	−9.1	0.37	**0.038**	0.242	0.382
- Activities of daily living	45.3 ± 13.2	48.0 ± 19.8	+5.9	1.97	**0.026**	46.6 ± 18.7	44.4 ± 17.3	−4.7	0.47	0.282	**0.004**	**0.015**
- Emotional well-being	59.4 ± 15.1	50.9 ± 24.6	−14.3	0.01	0.833	57.4 ± 14.7	45.6 ± 15.3	−20.6	2.15	**<0.0001**	**0.039**	**0.006**
- Stigma	47.7 ± 19.2	41.1 ± 18.1	−13.8	0.13	0.680	44.6 ± 20.8	35.4 ± 13.1	−20.6	1.63	**0.001**	0.056	0.064
- Social support	54.2 ± 28.9	43.1 ± 28.8	−20.5	0.04	0.752	40.7 ± 19.9	38.2 ± 19.3	−6.1	0.51	0.408	0.553	0.286
- Cognition	48.4 ± 17.2	51.6 ± 23.0	+6.6	0.30	0.394	50.9 ± 14.9	46.0 ± 15.4	−9.6	1.07	**0.026**	0.082	0.111
- Communication	43.8 ± 13.2	44.1 ± 21.2	+0.6	0.41	0.490	49.3 ± 20.7	40.5 ± 15.9	−17.8	2.17	**0.002**	**0.027**	**0.049**
- Bodily discomfort	57.3 ± 26.9	48.3 ± 25.5	−15.7	0.83	0.167	64.1 ± 20.6	55.8 ± 18.9	−12.9	1.13	**0.029**	**0.036**	**0.045**
**Secondary variables**												
BDI-II	12.7 ± 7.9	12.6 ± 9.6	−0.8	0.98	0.794	13.7 ± 7.3	11.9 ± 6.1	−13.1	1.52	**0.001**	0.650	0.484
NMSS	56.0 ± 41.9	49.8 ± 38.4	−11.1	0.11	0.051	47.2 ± 31.4	37.6 ± 28.0	−20.3	0.13	**0.046**	0.600	0.624
ADLS	77.5 ± 20.7	81.0 ± 9.1	+4.5	0.04	0.755	80.0 ± 17.1	81.3 ± 13.6	+1.6	0.04	0.829	0.619	0.668
EUROHIS-QOL8	3.7 ± 0.7	3.6 ± 0.9	−0.1	0.02	0.470	3.7 ± 0.8	3.7 ± 0.6	0	0.03	0.539	0.312	0.989

The results represent mean ± SD; RC (%), relative change; ES, Cohen’s d effect size; p^1^, comparison between V6 and V0 after using the Wilcoxon signed-rank test in the group without intervention (PwP-C); p^2^, comparison between V6 and V0 after using the Wilcoxon signed-rank test in the group with intervention (PwP-T); p^3^, comparison between V6 and V0 after using repeated-measures ANOVA without adjustment according to the intervention (PwP-C vs. PwP-T); p^4^, comparison between V6 and V0 after using repeated-measures ANOVA with adjustment for covariates (age, sex, and disease duration) according to the intervention (PwP-C vs. PwP-T). In bold, *p* < 0.05 (statistical significance). Nominal significance was defined as *p* < 0.05; statistical significance after Bonferroni correction was defined as *p* < 0.00625. ADLS, Schwab and England Activities of Daily Living; BDI-II, Beck Depression Inventory II; EUROHIS-QOL8, EUROHIS 8-item questionnaire; NMSS, Non-Motor Symptoms Scale; PDQ-39, 39-item Parkinson’s Disease Quality of Life Questionnaire; PwP, people with Parkinson’s disease; PwP-C, PwP control group; PwP-T, PwP treatment group.

**Table 3:** Correlation between the change from V0 to V6 (Δ) in the PDQ-39 total score and the changes from V0 to V6 (Δ) in the other scores (BDI-II, NMSS, ADLS, EUROHIS-QOL8) in both groups, PwP-C (N = 24) and PwP-T (N = 36).

	PwP-C (N = 24) Δ PDQ-39	PwP-T (N = 36) Δ PDQ-39
Δ BDI-II	r = 0.683 ***p* < 0.0001**	r = 0.609 ***p* < 0.0001**
Δ NMSS	r = 0.340 *p* = 0.121	r = 0.506 ***p* = 0.003**
Δ ADLS	r = −0.139 *p* = 0.570	r = −0.562 ***p* = 0.001**
Δ EUROHIS-QOL8	r = −0.252 *p* = 0.312	r = −0.486 ***p* = 0.005**

Spearman correlation was used for all analyses except the correlation between Δ ADLS and Δ PDQ-39 due to the non-normal distribution of Δ ADLS. In bold, *p* < 0.05 (statistical significance). ADLS, Schwab and England Activities of Daily Living; BDI-II, Beck Depression Inventory II; EUROHIS-QOL8, EUROHIS 8-item questionnaire; NMSS, Non-Motor Symptoms Scale; PDQ-39, 39-item Parkinson’s Disease Quality of Life Questionnaire; PwP, people with Parkinson’s disease; PwP-C, PwP control group; PwP-T, PwP treatment grouped

## Data Availability

The protocol and the statistical analysis plan are available on request. Deidentified participant data are not available for legal and ethical reasons.

## Abbreviations

The following abbreviations are used in this manuscript:

ADLS	Schwab and England Activities of Daily Living Scale
BDI-II	Beck Depression Inventory-II
EUROHIS-QOL8	EUROHIS 8-item questionnaire
H&Y	Hoehn and Yahr stage
NMS	Non-motor symptoms
NMSS	Non-Motor Symptoms Scale
OL-CCTP	Online comprehensive care therapy program
PD	Parkinson’s disease
PDQ-39	39-Item Parkinson’s Disease Questionnaire
PwP	People with PD
QoL	Quality of life
